# Healthcare workers' experience when using an electronic medical order entry and bar-code technology in an ICU

**DOI:** 10.1186/cc11124

**Published:** 2012-03-20

**Authors:** R Fumis, I Souza, V Pizzo, G Schettino

**Affiliations:** 1 Hospital Sírio-Libanês, São Paulo, Brazil

## Introduction

Medication errors are frequent in the ICU and may occur during medical ordering, transcription or administration of drugs. A system consisting of a computerized physician order entry (CPOE) with bar-code verification of medications (TASY; Web Sistemas, Brazil) has been described as a tool to improve medication safety [1], but few data are available about the satisfaction of healthcare workers with the use of this new technology in the ICU.

## Methods

We conducted a survey to evaluate the satisfaction of healthcare workers when using a CPOE with bar-code verification of medications in a tertiary 40-bed adult ICU in Sao Paolo, Brazil 6 months after implementing the system. A satisfaction questionnaire which consisted of items in a numeric scale type from 1 (low satisfaction) to 10 (high satisfaction) was filled out by physicians (*n *= 42), nurses (*n *= 58), nurses technicians (*n *= 84) and other professionals (*n *= 66).

## Results

Most subjects were female (66%), below 36 years of age (69%) and used the computer daily at home (81%). On average, respondents were satisfied with the CPOE system (score 5.74 ± 2.14) and believed it improved safety (score 7.64 ± 2.42). Satisfaction was lower among physicians (score 4.62 ± 1.79) when compared to other professionals (score 5.97 ± 2.14; *P *< 0.0001). The ease to place the first medical order and to copy the order form the previous day scored 5.41 ± 2.05 and 6.39 ± 1.93. The visualization of the medical order with the bar-code verification of drugs administration scored 5.95 ± 2.51 by the nurses. On average, physicians found the system less user-friendly (score 3.88 ± 1.85) than other professionals (6.40 ± 2.29; *P *< 0.0001).

## Conclusion

Although most of the ICU staff believe that the CPOE and bar-code has the potential to improve medication safety and the quality of care for critically ill patients, our survey showed a low level of satisfaction 6 months after implementing the system, particularly for physicians who consider the system unfriendly.
